# Hospital Cost and Hospital Statistics

**Published:** 1915-12-25

**Authors:** 


*To the Editor of* The Hospital.

Assistant Secretary.

### Hospital Cost and Hospital Statistics.

*To the Editor of* The Hospital.

Sir,—You " slate " poor " Assistant Secretary " for writing that the public have no interest in hospital accounts. But it is none the less true, and the elaborate statistics, which are a pain and grief to the secretaries and accountants, are only useful to hospital managers and secretaries, and not by any means to all of them.

I do not remember a single instance in all my hospital career of being asked by a subscriber or by one of the public any question about the accounts. Sometimes I get letters fearing that " hospitals are very extravagantly managed," and then one has the reply that no other charities in England are so severely policed. No other charities have three "Funds" watching them and an "Annual" publishing their accounts. No other charities have to keep their accounts on a Uniform System for the purpose of comparison. And I am not sure that it is not just as well that the public should be so complacent. If they did criticise, the main charge against this or that hospital would be that it spent more per bed than another of the same size.

But is this "cost per bed" a fair basis of criticism? You do not condemn one newspaper because it spends more on its foreign correspondence than another of the same size. The test is not how much is spent per bed, but what work the hospital is getting done for its expenditure. For instance, if Hospital A has Finsen light, Tyrnauer baths, sulphur baths, a large Röntgen-ray establishment, salvarsan beds, a massage department, etc., and Hospital B has none of these things, is it fair to hold up Hospital B as a paragon of good management, and to condemn Hospital A because it spends more per bed? If Hospital B keeps its patients twenty-eight days in the wards, and Hospital A gets them out in nineteen, and so uses its beds to the greater public advantage, is it fair to condemn Hospital A because this is more expensive? To a non-expert "cost per bed" is perhaps the only test till we get an "efficiency" judge, but it is a very unsatisfactory test, and, what is worse, it is a very discouraging one to anybody who cares for progress. No progress would be possible if too much attention were paid to this test.

I remember ten years ago somebody in the *Times* drew attention to the great cost of London hospitals as compared with those of Glasgow. We sent up to Glasgow a small commission of experts, and what did we find?

At one hospital that had been specially held up as being so wonderfully cheaply managed—a model to copy—we found that only half the number of nurses were employed per bed that we employed at the "London." (Did this mean equally good nursing?) We found that two adults were in many of the beds—605 patients in 580 beds. The nurses had one day off a month, no substitutes were provided, and our commissioners were told that "if they could not arrange their work beforehand it was bad management on their part." The candidates had to pay £5 5s. for the preliminary course of training (three months), and to provide their own board and lodging in the district! The patients had roast meat only once a fortnight, and were allowed one good-sized potato when in season, and when not, bread instead. No puddings were served to them. Once a week the nurses were allowed eggs and bacon for breakfast, at the rate of half an egg a nurse (scrambled egg, I should think!). On the day our commissioners were there the nurses had tomato soup, mutton, and milk pudding for dinner. The tomato soup was made from tinned tomatoes, and cost 5s. 6d. for 150 nurses! and so on.

This may be all changed now. I hope so. But it does show how illusory the "cost per bed" test is, and the danger of inviting the public to make that the test. But what other can take its place?—Yours faithfully,

Knutsford.

[Lord Knutsford brings out in a few words the whole doctrine preached in our articles on the King's Fund statistics, that figures must not be allowed to become a master, but should be welcomed as a help, to be used side by side with essential information concerning the individual hospital.—Ed. The Hospital.]

### Kitchen and Stores Department: "Tip and Non-Tip" Kitchen Boilers.

*To the Editor of* The Hospital.

